# Effects of Iloprost on Oxygenation during One-Lung Ventilation in Patients with Low Diffusing Capacity for Carbon Monoxide: A Randomized Controlled Study

**DOI:** 10.3390/jcm11061542

**Published:** 2022-03-11

**Authors:** Kyuho Lee, Young Jun Oh, Mina Kim, Sei Han Song, Namo Kim

**Affiliations:** 1 Department of Anesthesiology and Pain Medicine, Yonsei University College of Medicine, Seoul 03722, Korea; theoneimlee@yuhs.ac (K.L.); yjoh@yuhs.ac (Y.J.O.); songseihan@yuhs.ac (S.H.S.); 2 Anesthesia and Pain Research Institute, Yonsei University College of Medicine, Seoul 03722, Korea; 3 Department of Anesthesiology and Pain Medicine, Dongguk University Ilsan Hospital, Goyang 10326, Korea; exrexr20@gmail.com

**Keywords:** one-lung ventilation, diffusing capacity for carbon monoxide, iloprost, oxygenation

## Abstract

The protective mechanism of hypoxic pulmonary vasoconstriction during one-lung ventilation (OLV) is impaired in patients with a low diffusing capacity for carbon monoxide (DL_CO_). We hypothesized that iloprost inhalation would improve oxygenation and lung mechanics in patients with low DL_CO_ who underwent pulmonary resection. Forty patients with a DL_CO_ < 75% were enrolled. Patients were allocated into either an iloprost group (ILO group) or a control group (*n* = 20 each), in which iloprost and saline were inhaled, respectively. The partial pressure of arterial oxygen/fraction of inspired oxygen (PaO_2_/FiO_2_) ratio, pulmonary shunt fraction, alveolar dead space, dynamic compliance, and hemodynamic parameters were assessed 20 min after the initiation of OLV and 20 min after drug administration. Repeated variables were analyzed using a linear mixed model between the groups. Data from 39 patients were analyzed. After iloprost inhalation, the ILO group exhibited a significant increase in the PaO_2_/FiO_2_ ratio and a decrease in alveolar dead space compared with the control group (*p* = 0.025 and *p* = 0.042, respectively). Pulmonary shunt, dynamic compliance, hemodynamic parameters, and short-term prognosis were comparable between the two groups. Selective iloprost administration during OLV reduced alveolar dead space and improved oxygenation while minimally affecting hemodynamics and short-term prognosis.

## 1. Introduction

One-lung ventilation (OLV) is required for operative procedures in the thoracic cavity. However, OLV aggravates ventilation–perfusion (V/Q) mismatch and commonly results in hypoxemia, which has an incidence of 5–10% [1]. Hypoxemia of the nonventilated lung triggers hypoxic pulmonary vasoconstriction (HPV), an autoregulatory mechanism that decreases the shunt fraction by diverting total pulmonary blood flow from the nonventilated lung to the ventilated lung [2,3].

The diffusing capacity for carbon monoxide (DL_CO_) measures the ability of the gas to diffuse across the alveolar–capillary membrane [4]. Reduced DL_CO_ is an independent risk factor for increased mortality and perioperative complications related to hypoxia [5,6]. The risk of hypoxia is further increased when patients with low DL_CO_ undergo surgeries requiring OLV because the protective mechanism of HPV is impaired owing to altered compliance of the pulmonary artery [7].

Pharmacological modulation of pulmonary perfusion to reduce V/Q mismatch is gaining interest, and inhaled iloprost is recognized to enhance oxygenation in patients with acute respiratory distress syndrome (ARDS), pulmonary arterial hypertension, and chronic obstructive pulmonary disease [8,9]. Yet, limited evidence exists regarding the use of iloprost in pulmonary resections, especially in patients with low DL_CO_. Hence, we hypothesized that despite impaired HPV in these patients, iloprost administration would reduce V/Q mismatch by inducing favorable modulation of pulmonary perfusion. This study aimed to investigate the effects of iloprost on oxygenation and lung mechanics in patients with low DL_CO_ who underwent OLV.

## 2. Materials and Methods

### 2.1. Study Population

This prospective, randomized controlled study included patients who were scheduled for videoscope-assisted thoracoscopic single pulmonary lobectomy between September 2015 and June 2017 and adhered to the applicable Consolidated Standards of Reporting Trials (CONSORT) guidelines. The study was approved by the Institutional Review Board (IRB, no. 4-2015-0706) of Severance Hospital, Yonsei University Health System (Seoul, Republic of Korea), and was registered at Clinicaltrials.gov (NCT 02784899). After IRB approval, informed consent was obtained from all subjects involved in the study, and the study methods were performed in accordance with the relevant guidelines and regulations. The inclusion criteria were as follows: (1) DL_CO_ < 75%, (2) age between 40 and 80 years, (3) American Society of Anesthesiologists physical status class between II and III. The exclusion criteria were heart failure (New York Heart Association class III or IV), anemia, arrhythmia, severe hepatic or renal disease, and history of chemotherapy or radiation therapy prior to the surgery. Anemia was defined as hemoglobin concentration <12.0 g/dL in women and <13.0 g/dL in men [10].

### 2.2. Anesthetic Management

Anesthesia was induced with propofol (1.0–2.0 mg/kg), remifentanil (0.5–1.0 μg/kg), and rocuronium (0.8–1.0 mg/kg). All patients were intubated with left-sided double-lumen tubes (Shiley double-lumen endobronchial tube (DLT); Covidien, Mansfield, MA, USA). The correct positioning of the DLT was confirmed using a fiberoptic bronchoscope before OLV was provided. The radial artery was cannulated, and a 7-Fr central venous catheter (Arrow; Teleflex Inc., Wayne, PA, USA) was placed in the right internal jugular vein. Mechanical ventilation was provided using autoflow pressure-controlled ventilation mode (Primus; Dräger Medical, Lubeck, Germany). Anesthesia was maintained with 1.0–2.0 vol% sevoflurane and 0.1–0.3 μg/kg/min remifentanil targeted at bispectral index (BIS VISTA; Aspect Medical Systems, Norwood, MA, USA) between 40 and 60. Intraoperatively, balanced crystalloids were administered at a rate of 3 mL/kg/h, and additional crystalloids were administered to compensate for blood loss. Vasoactive drugs, such as ephedrine, were administered if systolic blood pressure (SBP) fell below 80 mmHg.

After turning a patient into the lateral decubitus position, OLV was initiated. The tidal volume was set at 6 mL/kg, and the inspiratory–expiratory ratio was set at 1:2. The fraction of inspired oxygen (FiO_2_) level was initially set at 0.6. In cases of desaturation (SpO_2_ < 95%), the FiO_2_ level was increased by 0.2, up to 1.0, and positive end-expiratory pressure (PEEP) of 5 mmHg was applied if SpO_2_ ≥ 95% was still not achieved.

### 2.3. Study Design and Outcome Measurements

All enrolled patients were allocated to the study groups using a randomized sequence, and the surgeon and anesthesiologist were blinded to the group allocation. Patients were randomly allocated to either an iloprost group (ILO group) or a control group. Twenty minutes after the initiation of OLV, iloprost (20 μg (2 mL), Ventavis; Bayer AG, Leverkusen, Germany) was administered to patients allocated to the ILO group. Iloprost was mixed with normal saline (3 mL) and aerosolized using an ultrasonic nebulizer (PARI BOY SX; PARI GmbH, Starnberg, Germany) connected to the inspiratory limb of the ventilator system. A comparable volume (5 mL) of normal saline was aerosolized to the patients in the control group. Interventional medications were administered for 20 min.

The study time points were as follows: (1) 20 min after the initiation of OLV in the lateral decubitus position (T1) and (2) 20 min after iloprost or normal saline administration (T2). During each study period, respiratory and hemodynamic parameters were recorded, and arterial and venous blood samples were collected. Respiratory parameters included FiO_2_, end-tidal carbon dioxide (etCO_2_), the ratio of partial pressure of arterial oxygen to FiO_2_ (PaO_2_/FiO_2_), partial pressure of arterial oxygen (PaO_2_), arterial oxygen saturation (SaO_2_), pulmonary shunt (Qs/Qt), alveolar dead space, and dynamic compliance. Hemodynamic parameters included heart rate, arterial blood pressure, and central venous pressure. A blood gas analyzer (GEM Premier 4000; Instrumentation Laboratory, Lexington, MA, USA) was used to assess hemoglobin (Hb), PaO_2_, SaO_2_, partial pressure of arterial carbon dioxide (PaCO_2_), partial pressure of venous oxygen (PvO_2_), and venous oxygen saturation (SvO_2_).

The shunt fraction (Qs/Qt) was calculated using the following formula: (Qs/Qt = (CcO_2_ − CaO_2_)/(CcO_2_ − CvO_2_), where CaO_2_ = (1.34 × Hb × SaO_2_) + (0.0031 × PaO_2_), CvO_2_ = (1.34 × Hb × SvO_2_) + (0.0031 × PvO_2_), and CcO_2_ = (1.34 × Hb) + (0.0031 × [FiO_2_ × (P_atm_ − P_H2O_) − PaCO_2_/RQ]), where CcO_2_, pulmonary capillary blood oxygen content; CaO_2_, arterial oxygen content; CvO_2_, venous oxygen content; P_atm_, atmospheric pressure (760 mmHg at sea level); P_H2O_, partial pressure of water (45 mmHg); RQ, respiratory quotient (0.8). The dead space ventilation was calculated according to the Hardman and Aitkenhead equation (1.135 × (PaCO_2_ − EtCO_2_)/PaCO_2_ − 0.005) [11]. Dynamic compliance was calculated using the following equation: [tidal volume/(plateau airway pressure-PEEP)]. The incidences of intraoperative hypotension (SBP < 80 mmHg) and hypoxia (SpO_2_ < 90%) were recorded. Short-term prognosis, including hospital stay, and postoperative complications, such as air leak requiring chest tube insertion, pneumonia, and in-hospital mortality, were assessed.

### 2.4. Statistical Analysis

The primary outcome was the change in PaO_2_/FiO_2_ at 20 min after iloprost inhalation (T2), and the secondary outcome was the change in other respiratory mechanics, such as alveolar dead space, shunt fraction, and dynamic compliance. A previous study reported that the standard deviation of the PaO_2_/FiO_2_ ratio was 60 mmHg for an inhaled-iloprost group [9]. A mean difference of 60 mmHg for the PaO_2_/FiO_2_ ratio between the ILO and control groups was considered clinically significant in the preliminary data for the first 10 patients after iloprost administration. Hence, 17 patients were required in each group with a power of 80% and a significance level of 0.05. Considering a 10% dropout rate, 20 patients were included in each group. The unpaired Student’s *t*-test was used to analyze continuous variables, and the Wilcoxon signed-rank test was used to analyze variables that did not meet normality. Chi-square or Fisher’s exact test was used to compare categorical variables between the groups. Repeated variables were analyzed using a linear mixed model with the group and time and the interaction between groups and time as a fixed effect. Post hoc analysis with Bonferroni correction for within-group comparisons versus T1 and between-group comparisons versus T2 was performed for multiple comparisons. The results were expressed as mean (standard deviation), median (interquartile range), or number (percentage). Statistical analyses were performed using SPSS 25.0 software (IBM Corp., Armonk, NY, USA), and *p* < 0.05 was considered statistically significant.

## 3. Results

**Figure 1:**
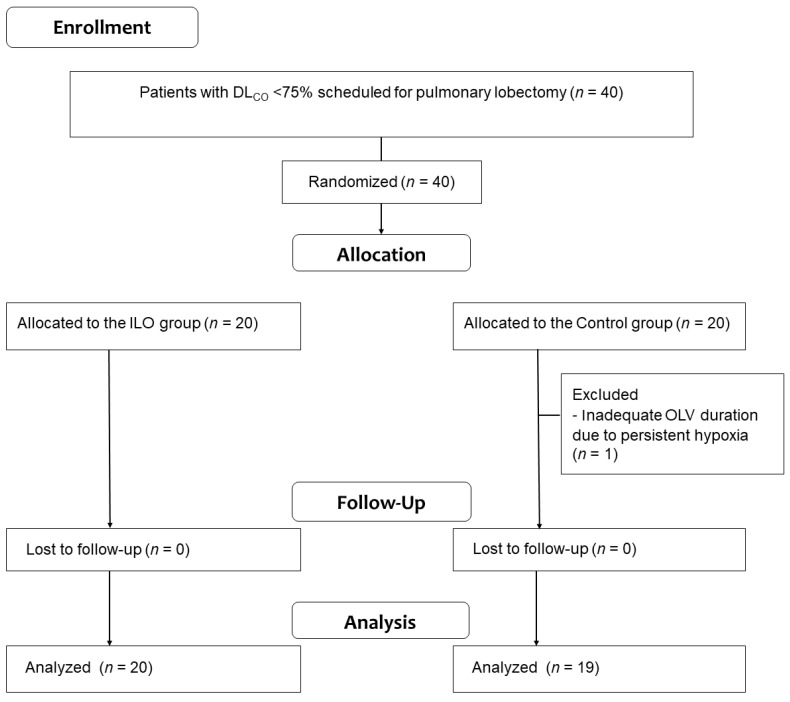
Patient enrollment.

Forty patients scheduled to undergo video-assisted thoracoscopic single pulmonary lobectomy were enrolled in this study. As OLV could not be achieved during the measurement period owing to persistent hypoxia in one patient, data from the remaining 39 patients were assessed (Figure 1).

Intergroup comparisons of the preoperative variables between the ILO and control groups are presented in Table 1. Age, sex, height, weight, body mass index, and ASA classification were comparable between the groups. Incidence of hypertension and diabetes mellitus, history of cigarette smoking, incidence of pulmonary abnormalities according to preoperative computed tomography, variables derived from preoperative spirometry, and DL_CO_ were also similar between the groups. None of the patients were associated with cardiac diseases such as heart failure.

**Figure 2:**
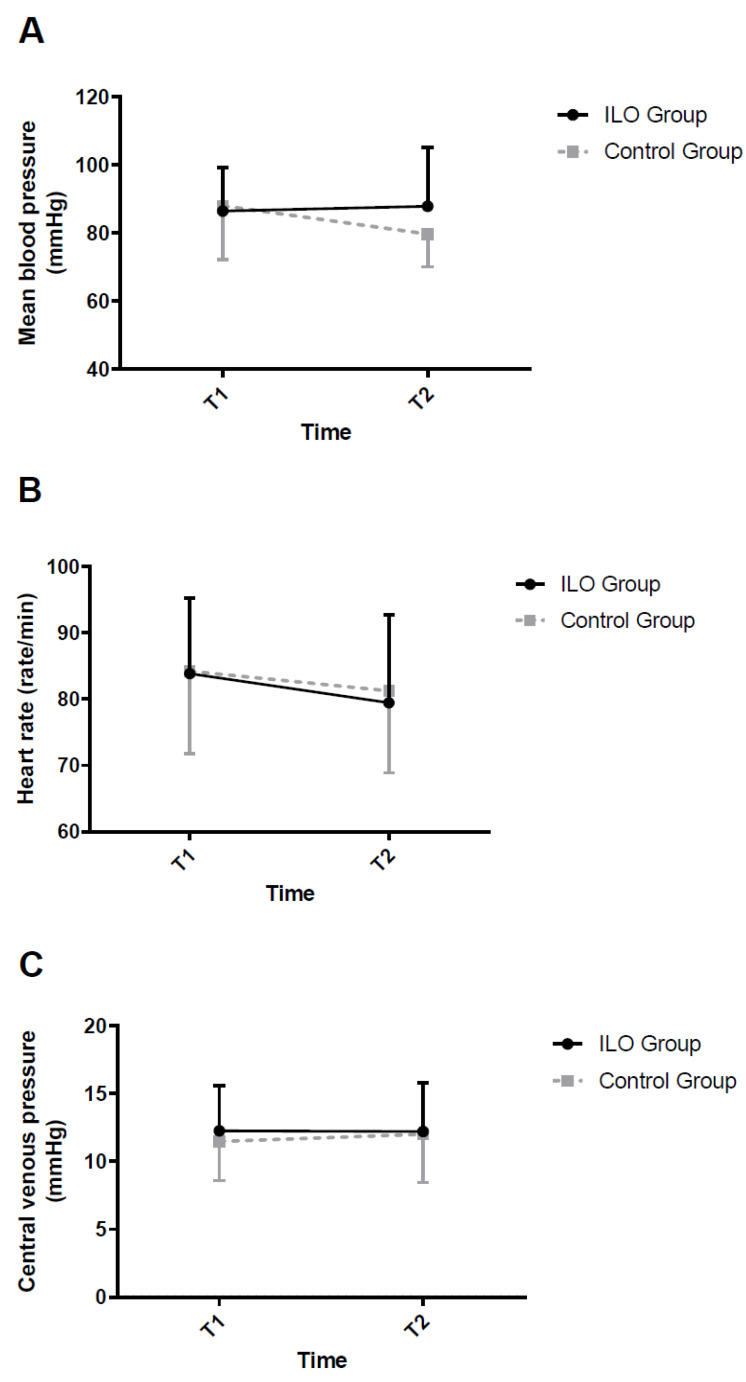
Effects of iloprost on hemodynamics. (**A**) Mean blood pressure, (**B**) heart rate, and (**C**) central venous pressure. Error bars represent standard deviation. No significant differences were observed between the two groups. T1, 20 min after initiation of one-lung ventilation in the lateral decubitus position; T2, 20 min after iloprost or saline administration.

Table 2 shows intergroup comparisons of the intraoperative data. All variables, including initial SpO_2_ measured at patients’ arrival at the operating room, side of the operation, anesthesia time, operation time, OLV time, incidence of intraoperative hypotension, intake fluid, urine output, and estimated blood loss during surgery, were comparable between the two groups with the exception of incidence of hypoxia requiring anesthetic intervention, which was more frequent in the control group (*p* = 0.031). Mean blood pressure, heart rate, and central venous pressure were also similar between the two groups (Figure 2).

The oxygenation parameters, lung mechanics, and hemodynamic data are shown in Table 3. No clinically relevant differences were observed between the two groups at T1. After iloprost administration, the ILO group showed a significant increase in the PaO_2_/FiO_2_ ratio, PaO_2_, and SaO_2_ and a decrease in alveolar dead space when compared with T1 (*p* = 0.044, *p* = 0.044, *p* = 0.024, and *p* < 0.001, respectively), which also resulted in significant differences compared with the control group. The pulmonary shunt at T2 was significantly decreased when compared with T1 in the ILO group (*p* = 0.014), but the difference compared with that of the control group was insignificant. Changes in dynamic compliance were insignificant among the groups.

The short-term prognosis of the patients is presented in Table 4. No significant differences in the duration of hospital stay or the incidence of postoperative complications, such as air leak, postoperative pneumonia, and in-hospital mortality, were observed between the two groups.

## 4. Discussion

In this study, we demonstrated that the selective administration of iloprost to ventilated lungs during OLV significantly reduced alveolar dead space and improved oxygenation in patients with low DL_CO_.

Reduced DL_CO_ is associated with the loss of alveolar membrane surface area and vascular remodeling, resulting in a reduced alveolar–capillary membrane diffusing capacity [12,13,14]. All patients exhibited mild to moderate decreases in DL_CO_ in the context of normal spirometry. A preoperative CT scan indicated early stages of diffuse interstitial lung disease or emphysema in most of the patients. Although all patients maintained SpO_2_ ≥ 95% at the end of the surgery, transient declines of SpO_2_ < 90% were observed in seven patients during OLV despite the application of increased FiO_2_ or PEEP. The incidence of hypoxia in our study surpassed that of healthy patients (18% vs. 5–10% [1]), indicating that impaired HPV, expressed as low DL_CO_ [7], aggravated V/Q mismatch during OLV.

The favorable effect of inhaled iloprost on oxygenation is well-described in patients with ARDS [15]. A similar mechanism would be favorable in patients undergoing OLV; however, evidence regarding the effect of iloprost administration in such a cohort is scarce. Choi et al. reported improved oxygenation and decreased intrapulmonary shunts with iloprost use in pulmonary resections; however, their study excluded patients with abnormalities in preoperative spirometry [16]. Our results suggested that consistent outcomes were observed in patients with low DL_CO_ and that iloprost significantly reduced alveolar dead space, which contributed to an increased PaO_2_/FiO_2_ ratio. In addition, the incidence of hypoxia was significantly less frequent in the ILO group.

However, contradictory results have been reported regarding inhaled iloprost and oxygenation [17,18]. A potential explanation is that the nonselective delivery of iloprost in awake patients may have led to conflicting results in those studies. We presume that to improve oxygenation using iloprost, the administration of the drug should be restricted to well-ventilated areas of the lung. Therefore, the use of a lung separation device, such as DLT, provides an ideal environment for iloprost administration in thoracic surgeries as it strictly confines the delivery of iloprost to the ventilated lung, which favorably redistributes the pulmonary perfusion from the nonventilated lung to the ventilated lung.

Concerns may arise regarding the safety of iloprost use in pulmonary resection. Although a previous study demonstrated that iloprost did not induce systemic adverse events in patients with ARDS [9], it may still be associated with a significant decrease in systemic blood pressure [19]. However, our results indicated that the incidence of intraoperative hypotension was comparable between the two groups. Another concern is that iloprost may be associated with the inhibition of platelet activation [9]; however, estimated blood loss was comparable between the two groups, and none of the patients in the ILO group required intraoperative transfusion. The incidence of postoperative complications and the duration of hospital stay were also similar between the two groups, which supports the notion that acute inhalation of iloprost (20 μg) is less likely to be associated with adverse events during the intraoperative and postoperative periods.

This study had some limitations. First, our patients rarely exhibited a decrease in DL_CO_ < 40% preoperatively, which limits the efficacy of inhaled iloprost in patients with a mild to moderate severity grade of low DL_CO_. However, because a very low DL_CO_ greatly increases the risk of morbidity and mortality after pulmonary resection [20,21], such patients are rarely introduced to the operating room. Second, the initiation of PEEP was delayed until hypoxia occurred despite elevated FiO_2_ to demonstrate the effect of iloprost because PEEP may compress the small interalveolar vessels of the ventilated lung [22], which hinders the vasodilation effect of iloprost and aggravates V/Q mismatch. Third, because a pulmonary artery catheter is not routinely used in single pulmonary lobectomy, we were unable to acquire blood samples from the pulmonary artery. Instead, right atrial blood samples were used to calculate the shunt fraction, although evidence supports that pulmonary arterial blood samples can be substituted for right atrial blood samples [23]. In addition, we could not exclude the presence of intraoperative pulmonary hypertension in the absence of the pulmonary artery catheter, although even a mild grade of chronic obstructive pulmonary disease may be associated with increased pulmonary arterial stiffness [7].

In conclusion, the results of this study support the use of iloprost inhalation as a possible rescue strategy against hypoxia during OLV. Selective iloprost administration during OLV reduced alveolar dead space and improved oxygenation while minimally affecting intraoperative hemodynamics and short-term prognosis in patients with low DL_CO_.

## Figures and Tables

**Table 1:** Preoperative data.

	ILO Group (*n* = 20)	Control Group (*n* = 19)	*p*-Value
Age (years)	68 ± 9	63 ± 10	0.173
Women (*n*)	10 (50)	6 (31.6)	0.242
Height (cm)	159.3 ± 10.4	164.2 ± 9.5	0.125
Weight (kg)	63.8 ± 11.9	68.1 ± 12.8	0.280
Body mass index (kg/m^2^)	24.7 ± 4.4	25.0 ± 3.6	0.674
ASA classification 2/3 (*n*)	11 (55)/9 (45)	9 (47.4)/10 (52.6)	0.634
Hypertension (*n*)	6 (30)	7 (37)	0.651
Diabetes mellitus (*n*)	4 (20)	4 (21)	0.935
Smoking history			0.113
Ex-smoker or current smoker (*n*)	11 (55)	15 (78.9)	
Nonsmoker (*n*)	9 (45)	4 (21.1)	
Smoking index (pack × years)	10 (0–50)	31 (3–41)	0.398
Preoperative chest CT			
Atelectasis (*n*)	2 (10)	0 (0)	0.157
Bronchiectasis (*n*)	2 (10)	0 (0)	0.157
Pleural effusion (*n*)	1 (5)	2 (11)	0.517
Emphysema (*n*)	10 (50)	7 (36.8)	0.408
Interstitial lung disease (*n*)	3 (15)	5 (26.3)	0.382
Preoperative spirometry			
FEV_1_ (L)	1.9 ± 0.6	2.6 ± 2.3	0.173
FEV_1_ (% predicted)	88.9 ± 20.7	83.3 ± 17.7	0.368
FVC (L)	2.8 ± 0.9	3.1 ± 1.0	0.320
FVC (% predicted)	89.5 ± 15.6	84.7 ± 19.0	0.388
FEV_1_/FVC (%)	69.6 ± 11.1	71.5 ± 13.6	0.517
DL_CO_ (% predicted)	65.5 ± 6.1	61.1 ± 10.6	0.117

Data are presented as mean ± standard deviation, number (%), or median (interquartile range). ASA, American Society of Anesthesiologists; CT, computed tomography; FEV_1_, forced expiratory volume in 1 s; FVC, forced vital capacity; DL_CO_, diffusion capacity of the lung for carbon monoxide.

**Table 2:** Intraoperative data.

	ILO Group (*n* = 20)	Control Group (*n* = 19)	*p*-Value
Initial SpO_2_ at room air (%)	98 (97–99)	96.0 (95–99)	0.918
Lobectomy (right/left) (*n*)	11 (55)/9 (45)	9 (47)/10 (53)	0.634
Anesthesia time (min)	200 (180–225)	183 (151–233)	0.473
Operation time (min)	138 (120–161)	118 (100–175)	0.336
OLV time (min)	115 (95–135)	103 (81–149)	0.603
Hypotension (*n*)	8 (40.0)	12 (63.2)	0.206
Hypoxia (*n*)	1 (5.0) *	6 (31.6)	0.031
Intake fluid (mL)	1315.8 ± 316.9	1454.0 ± 581.3	0.356
Urine output (mL)	241.3 ± 161.1	256.4 ± 152.3	0.768
Estimated blood loss (mL)	102.5 ± 63.8	136.0 ± 110.6	0.248

Data are presented as mean ± standard deviation, number (%), or median (interquartile range). SpO_2_, oxygen saturation (pulse oximetry); OLV, one-lung ventilation; hypotensive event defined as the incidence of systolic blood pressure < 80 mmHg; hypoxic event defined as the incidence of SpO_2_ < 90% requiring anesthetic intervention. * *p* < 0.05 vs. control group.

**Table 3:** Effects of iloprost on hemodynamics, oxygenation, and lung mechanics.

	ILO Group (*n* = 20)	Control Group (*n* = 19)	*p*-Value
FiO_2_			0.157
T1	0.6 (0.6–0.9)	0.6 (0.6–0.7)	
T2	0.6 (0.6–0.8)	0.8 (0.6–0.8)	
PaO_2_/FiO_2_ ratio (mmHg)			0.025
T1	125.9 (100.1–222.0)	138.3 (110.0–191.7)	
T2	141.4 (120.8–247.7) *^†^	128.3 (100.0–161.8)	
PaO_2_ (mmHg)			0.044
T1	84.8 (70.3–139.7)	83.0 (74.0–116.0)	
T2	104.7 (82.3–148.6) *^†^	81.0 (81.0–110.3)	
SaO_2_ (%)			0.026
T1	95.0 (92.8–98.5)	94.2 (92.8–97.3)	
T2	97.1 (95.5–99.8) *^†^	95.3 (92.2–97.3)	
Pulmonary shunt (%)			0.027
T1	27.0 ± 17.9	25.1 ± 17.8	
T2	18.4 ± 11.8 *	26.6 ± 14.4	
Alveolar dead space			0.042
T1	16.4 ± 5.0	19.2 ± 11.6	
T2	10.8 ± 7.3 *^†^	19.2 ± 11.0	
Dynamic compliance (mL/cm H_2_O)			0.055
T1	20.0 ± 5.3	21.4 ± 4.7	
T2	21.5 ± 7.9	20.2 ± 4.5	

Data are presented as the median (interquartile range) or mean ± standard deviation. FiO_2_, fraction of inspired oxygen; T1, 20 min after initiation of OLV (one-lung ventilation) in the lateral decubitus position; T2, 20 min after iloprost or saline administration; PaO_2_/FiO_2_ ratio, the ratio of partial pressure of arterial oxygen to FiO_2_; PaO_2_, partial pressure of arterial oxygen; SaO_2_, arterial oxygen saturation. Group × time, linear mixed model analysis as a random effect; and group, time, and group-by-time as fixed effects, * *p* < 0.05, vs. T1; ^†^ *p* < 0.05, vs. control group.

**Table 4:** Short-term prognosis.

	ILO Group (*n* = 20)	Control Group (*n* = 19)	*p*-Value
Hospital days	6 (5–8)	7 (4–9)	0.540
Postoperative complications	3 (15.0)	6 (31.6)	0.219
Air leak	1 (5.0)	3 (15.8)	0.267
Pneumonia	2 (10.0)	4 (21.1)	0.339
In-hospital mortality	1 (5.0)	2 (5.3)	0.970

Data are presented as median (interquartile range) or number (%).

## Data Availability

Data are available from the corresponding author upon reasonable requests.
